# The Two Faces of the Guanyl Radical: Molecular Context and Behavior

**DOI:** 10.3390/molecules26123511

**Published:** 2021-06-09

**Authors:** Chryssostomos Chatgilialoglu

**Affiliations:** 1 ISOF, Consiglio Nazionale delle Ricerche, 40129 Bologna, Italy; chrys@isof.cnr.it; Tel.: +39-051-6398309; 2 Center of Advanced Technologies, Adam Mickiewicz University, 61-712 Poznań, Poland

**Keywords:** guanine, guanyl radical, tautomerism, guanine radical cation, oligonucleotides, DNA, G-quadruplex, time-resolved spectroscopies, reactive oxygen species (ROS), oxidation

## Abstract

The guanyl radical or neutral guanine radical G(-H)^•^ results from the loss of a hydrogen atom (H^•^) or an electron/proton (e^–^/H^+^) couple from the guanine structures (G). The guanyl radical exists in two tautomeric forms. As the modes of formation of the two tautomers, their relationship and reactivity at the nucleoside level are subjects of intense research and are discussed in a holistic manner, including time-resolved spectroscopies, product studies, and relevant theoretical calculations. Particular attention is given to the one-electron oxidation of the GC pair and the complex mechanism of the deprotonation vs. hydration step of GC^•+^ pair. The role of the two G(-H)^•^ tautomers in single- and double-stranded oligonucleotides and the G-quadruplex, the supramolecular arrangement that attracts interest for its biological consequences, are considered. The importance of biomarkers of guanine DNA damage is also addressed.

## 1. The Guanine Sink

The free radical chemistry associated with guanine (Gua) and its derivatives, guanosine (Guo), 2’-deoxyguanosine (dGuo), guanosine-5′-monophosphate (GMP), and 2′-deoxyguanosine-5′-monophosphate (dGMP), is of particular interest due to its biological relevance. Indeed, this reactivity is by far the most involved in oxidative DNA damage, initiated by reactive oxygen species (ROS) generated by normal cellular metabolism, or by exogenous sources such as ionizing or ultraviolet irradiation [1,2,3]. In the thirty years since the discovery of long-range charge transport in DNA, as reviewed by Barton and coworkers [4,5,6], a large body of experimental data have accumulated showing that the one-electron oxidation of DNA produces a hole that can migrate through the double helix with the final destination at the G sites [4,5,6,7,8,9,10]. Among the four common DNA bases (A, G, T, and C), G is the most readily oxidized to the G radical cation (G^•+^), which is also the putative initial intermediate in the oxidative DNA damage. Thus, there have been numerous studies on the formation and behavior of G^•+^ as a nucleoside and in oligonucleotides (ODNs) using spectroscopic and product studies. Upon formation of G^•+^, fast deprotonation occurs by the loss of a proton to give the guanyl radical G(-H)^•^, also named the guanine radical or neutral guanine radical. The main objective of this review is to summarize the behavior of G(-H)^•^ in nucleosides and nucleotides and give an overview of its formation in a macromolecular environment, such as single-stranded (ss), double-stranded (ds) ODNs, and G-quadruplex arrangements.

## 2. The Two Tautomers of the Guanyl Radical

**Figure 1:**
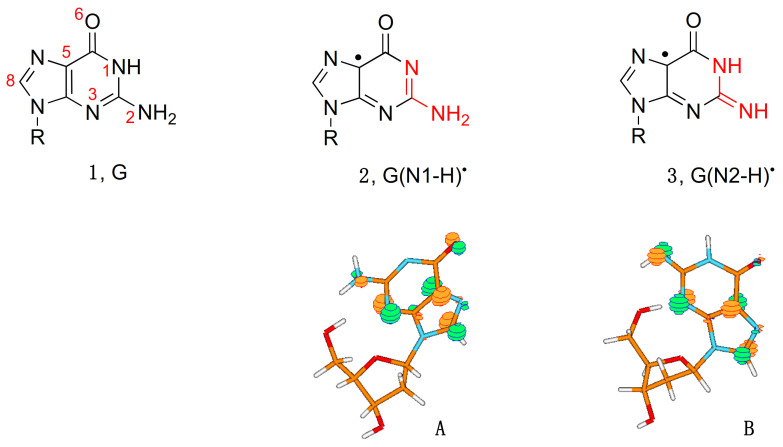
Structure **1** represents the guanine moiety in guanosine (Guo), 2′-deoxyguanosine (dGuo), guanosine-5′- monophosphate (GMP), 2′-deoxyguanosine-5′-monophosphate (dGMP), or 3′,5′-cyclic guanosine monophosphate (cGMP); the atom numbering of the guanine moiety is given in red. The corresponding guanyl radicals G(N1-H)^•^ (**2**) or G(N2-H)^•^ (**3**); the part of the molecule involved in tautomerization is highlighted in red. SOMO of the two forms of guanyl radical G(N1-H)^•^ (**A**) and G(N2-H)^•^ (**B**) computed at the B3LYP/6-311G** level (taken from ref [11]).

The guanyl radical G(-H)^•^ corresponds to any intermediate that has lost an H-atom or an electron/proton (e^–^/H^+^) couple from the G moiety. G(-H)^•^ is one of the most recurrent and intensively studied transient species. The reason is obvious, as dGuo (**1**, R = 2′-deoxyribose) is one of the building blocks of DNA with the lowest oxidation potential (Figure 1). The chemistry of G(-H)^•^ depends on the molecular context where it belongs (free base, nucleoside in ribo or deoxyribo forms, ss- or ds-ODNs including multi-G sequences, RNA, DNA, and G-quadruplex). Although numerous mechanistic studies have been performed during recent years, the emerging picture is still incomplete, with several unanswered questions and divergent opinions. The extrapolation of its chemistry from simple nucleosides to more complex environments (e.g., DNA) can be misleading. This situation often generates confusion to the newcomers in the field. In the present article, the existing dichotomies among mechanistic insights are examined and critically discussed to give a reasoned overview of the guanyl radical structure and behavior.

Figure 1 shows two tautomeric structures of G(-H)^•^: form **2** or G(N1-H)^•^ that has lost H from the N1 position, and form **3** or G(N2-H)^•^ that has lost H from the N2 position. The acronyms G(N1-H)^•^ and G(N2-H)^•^ have been commonly used in the recent literature and are used throughout this review; alternative acronyms have been used: G^•^, N1G^•^, (G-H_1_)^•^ or the aminic form for G(N1-H)^•^, and *N*(2)G^•^, N2G^•^, (G-H_2_)^•^, G(*N*^2^-H)^•^ or the iminic form for G(N2-H)^•^. Figure 1 also shows the distribution of the unpaired electron in the SOMO of G(N1-H)^•^ (**A**) and G(N2-H)^•^ (**B**) computed at the B3LYP/6-311G** level [11]. In **A**, the unpaired electron is mainly localized at the N3, O6, C5, and C8 atoms (spin densities of 0.30, 0.34, 0.21, and 0.27, respectively), whereas, in **B**, the unpaired electron is mainly localized at the N3, C5, C8, and N2 atoms (spin densities of 0.34, 0.29, 0.22, and 0.39, respectively) [11]. As each tautomer has various resonance forms (discussed in some detail in reference [11]), the reported drawings for radicals **2** and **3**, where the unpaired electron is placed in the C5 position, are chosen for a facile distinction of the two tautomers (Figure 1). 

## 3. One-Electron Oxidation of Guanine Derivatives

**Figure 2:**
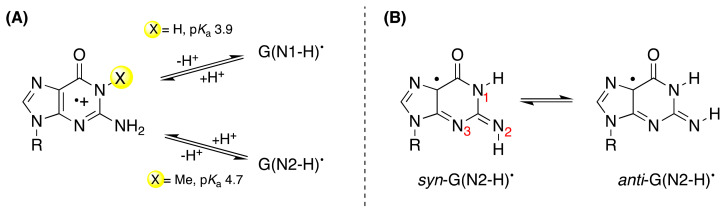
(**A**) The deprotonation of G^•+^ (X = H) and 1-Me-G^•+^ (X = Me) affording G(N1-H)^•^ and G(N2-H)^•^, respectively. (**B**) Conformational isomers of G(N2-H)^•^ in *syn* and *anti* with respect to N3 atom; the earlier *syn*- and *anti*-conformations were defined with respect to N1-H [11] and, as *syn* and *anti* with respect to N3 have been commonly used in the recent literature, these notations are adopted in the text.

In the guanine derivatives **1** (G), the two p*K*_a_ values of 2.5 and 9.3 are related to the protonation and deprotonation steps, respectively [12,13]. Early work by pulse radiolysis studies with both conductometric and optical detection showed that the one-electron oxidations of Guo and dGuo by the powerful SO_4_^•−^ or the milder Br_2_^•−^ oxidants (see Appendix A) afford the corresponding radical cation G^•+^, which is in agreement with the reduction potentials of the reactants E_7_(**2**/**1**) = 1.29 V, *E*°(SO_4_^•−^/SO_4_^2−^) ≈ 2.43 V, and *E*°(Br_2_^•−^/2Br^−^) = 1.66 V [12,14,15]. The determined p*K*_a_ value of 3.9 in G^•+^ is associated with the loss of a proton from N1 to give the guanyl radical G(N1-H)^•^ (Figure 2); at high pH, G(N1-H)^•^ further deprotonates to produce G(-2H)^•−^ with p*K*_a_ 10.7 [12,13]. Therefore, the one-electron oxidation of **1** in the pH range 5–8 affords the guanyl radical G(N1-H)^•^. The deprotonation rate constant was measured at pH 7 by pulse radiolysis to be 1.8 × 10^7^ s^−1^ at r.t., showing a *k*(H_2_O)/*k*(D_2_O) of 1.7 [16] and by laser flash photolysis to be 1.5 × 10^7^ s^–1^ with the activation energy of 15.1 ± 1.5 kJ mol^–1^ [17]. DFT calculations on solvation models were used to calculate the deprotonation potential energy profile [17]. The one-electron oxidation of Guo by Br_2_^•−^ was investigated by pulse radiolysis coupled with transient electrochemistry; the intermediate G(N1-H)^•^ (at 28 ± 4 μM concentration) decays by a second-order rate of 3.4 × 10^7^ M^−1^ s^−1^, and there is competition between the oxidation and reduction of G(N1-H)^•^, i.e., the ability of the radical to be either oxidized or reduced, presumably via disproportionation rather than the dimerization process [18].

On the other hand, when the N1-H moiety is replaced by N1-Me, the deprotonation occurs from the exocyclic NH_2_ group and associates with a p*K*_a_ value of 4.7 (Figure 2A) [14]. Indeed, theoretical calculations have suggested that deprotonation from the N2-H moiety is competitive with deprotonation from N1-H. Both EPR and UV-visible spectra of the one-electron-oxidized dGuo by Cl_2_^•−^ are reported for each of the species: G^•+^, G(-H)^•^, and G(-2H)^•−^ in aqueous medium at 77 K [19]. The experimental data corroborated by DFT calculations indicated that G(N1-H)^•^ is favored over G(N2-H)^•^ in environments with high dielectric constants, such as water. Furthermore, the two conformational isomers of G(N2-H)^•^ in which the remaining N2–H is either *syn* or *anti* with respect to the guanine N3 atom were calculated, finding that the *syn*-conformer is lower in energy that the *anti*-conformer is (Figure 2B) [19]; see also the three-dimensional structure **B** in Figure 1 that refers to the *syn*-conformer [11].

The oxidation of GMP and dGMP has also been reported in some detail. It has been reported that the presence of the phosphate group in dGMP^•+^ does not significantly affect the prototropic equilibria of the guanine moiety. ENDOR studies carried out using X-ray-irradiated single crystals of dGMP showed that the deprotonation of G^•+^ occurs from N2 rather than the N1 position with the formation of G(N2-H)^•^ [20]. More recently, the structure and reactivity of G^•+^ and G(-H)^•^ have been investigated in aqueous solutions by using pulse radiolysis (Raman and UV−visible detection) from the 5′-dGMP oxidation by SO_4_^•−^ at pH 7.4 [21], or by the photo-oxidation of 5′-GMP by the 3,3′,4,4′-benzophenone tetracarboxylic acid (TCBP) triplet within the pH range of 2−12 using laser flash photolysis and time-resolved chemically induced dynamic nuclear polarization (CIDNP) [22,23,24]. There is strong spectroscopic evidence that G(N1-H)^•^ is further transformed to a new species in the range of pH 7.4–8.0 [21,23,24]. The formation of this species shows a pH dependence, suggesting that it is the G radical cation, named (G^•+^)′, and is tentatively assigned to the protonation at the N7 position of G(N1-H)^•^ with a rate constant of 8.1 × 10^6^ s^−1^. Although this suggestion is strongly criticized based on theoretical calculations [25], further support is provided by time-resolved CIDNP [23,24]. From the full analysis of the pH-dependent CIDNP kinetics, the protonation and deprotonation behavior was quantitatively characterized, giving p*K*_a_ = 8.0 ± 0.2 of the radical (G^•+^)′ [24]. The same spectroscopical method suggested the formation of the radical dication G^•++^ with p*K*_a_ = 2.1 [22]. The above-described experiments are based on the oxidation of GMP or dGMP and the corresponding guanyl radical. It is well-known that GMP and dGMP with respect to Guo and dGuo have two extra p*K*_a_ values of 0.3 and 6.3 that correspond to the deprotonation of the phosphate moiety, viz. P(O)(OH)_2_ and P(O)(OH)O^−^ [26]. Although the observed p*K*_a_ values around 2 and 8 could be associated with the deprotonation of the phosphate moiety in the one-electron oxidation of GMP or dGMP, in the earlier report, the authors mentioned that the results are qualitatively the same by replacing GMP with Guo [23]. More detailed analysis on Guo is necessary to clarify this aspect.

## 4. One-Electron Reduction of 8-Haloguanine Derivatives

**Figure 3:**
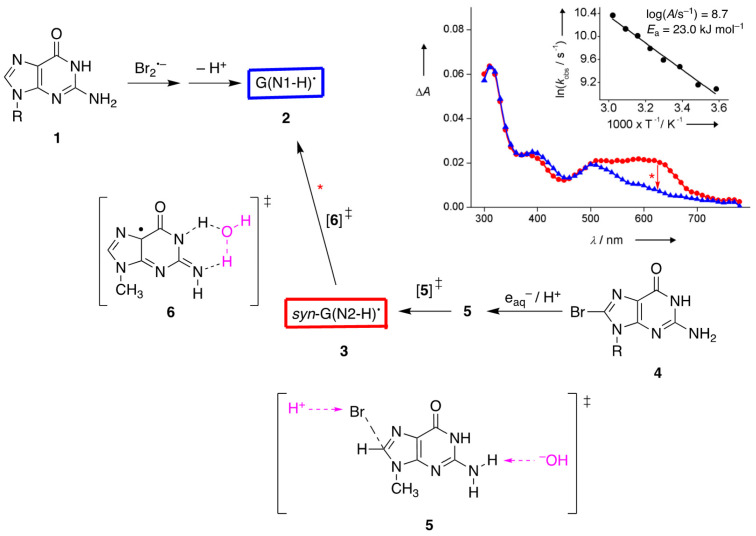
Oxidation of guanosine **1** by Br_2_^•−^ and the formation of transient guanyl radical **2** (in blue color). The reduction of 8-bromoguanosine **4** by e_aq_^−^ and the transient guanyl radical **3** taken in 2 μs after the pulse (in red color); by monitoring the reaction at 620 nm (see red arrow), radical **3** is transformed to **2** with a first-order rate constant of 5 × 10^4^ s^−1^ at room temperature with Arrhenius parameters reported in the insert (adapted from [11,28]). The transition state **5**, for the deprotonation from NH_2_ by means of HO^−^ during the elimination of Br^–^ from the π-complex as HBr, and the transition state **6**, for the H_2_O-assisted tautomerization, were calculated at the PCM/B1B95/6-31+G**//B1B95/6-31+G** level.

The first directly observed differences of the two tautomeric forms of G(-H)^•^ came from a pulse radiolysis study. Using the reaction of hydrated electrons (e_aq_^−^) with 8-haloguanosine (halo = Cl, Br, and I), the two observed short-lived intermediates, which show a substantial difference in their absorption spectra around 620 nm, were assigned to the two G(-H)^•^ tautomers [11]. The identification of various transient species and transition states has also been addressed computationally by means of time-dependent DFT (TD-B3LYP/6-311G**//B1B95/6-31+G**) calculations [11]. Figure 3 shows the transient UV-visible spectra of the reaction of 8-Br-Guo (**4**) with e_aq_^−^ to produce the less stable G(N2-H)^•^ (in red color), followed by a tautomerization to give G(N1-H)^•^ (in blue color), for which an identical spectrum was obtained by the one-electron oxidation of Guo (**1**). Theory suggests that the electron adduct of 8-Br-Guo protonated at C8 forms a loose π-complex with the Br atom situated above the molecular plane, and is prompt to eject Br^−^. Moreover, the transition state **5** for the elimination of *anti*-H from the NH_2_ group was found to be much more stable than the transition states for eliminations of *syn*-H by 18.0 kJ mol^−1^ and of N1-H by 14.1 kJ mol^–1^. Presumably, the observed spectrum is the *syn*-G(N2-H)^•^ radical [11]. The band around 620 nm was also predicted very well by the DFT-TD calculations [27], i.e., λ = 616 nm, *f* = 0.045, and transition {spin}: pπ(out-of-phase N2′, N1, O6′) ⟶ SOMO (out-of-phase N2′, N3, C5, C8) {b}.

The Arrhenius parameters for this tautomerization, the solvent kinetic isotope effect (*k*(H_2_O)/k(D_2_O), and the calculations at the PCM/B1B95/6-31+G**//B1B95/6-31+G** level suggested a complex transition state **6** for the water-assisted tautomerization [11,28,29]; the reaction mechanism of e_aq_^−^ with **4** reported in 2000 [29] was revised in 2005 [28], and the revision did not affect the experimental results (the kinetic data). For the ribose series, the rate constants for the transformation G(N2-H)^•^ ⟶ G(N1-H)^•^ were measured in H_2_O and D_2_O, *k*(H_2_O) = 5.0 × 10^4^ s^−1^ and *k*(D_2_O) = 6.2 × 10^3^ s^−1^, respectively, with a solvent kinetic isotope effect of 8.0 [29]. The Arrhenius parameters, log(*A*/s^−1^) = 8.7 ± 0.4 and *E*_a_ = 23.0 ± 2.5 kJ mol^−1^, were measured in the temperature range of 5.8–50.3 °C [28]. The tautomerization is accelerated by phosphate and H^+^, with the rate constants *k*_phosp_ = 1.4 × 10^8^ M^−1^ s^−1^ at pH~7 and k_H+_ ≅ 1 × 10^10^ M^−1^ s^−1^ at pH 4, respectively [29]. The G(N2-H)^•^ is unreactive toward methyl viologen (MV2+) and molecular oxygen, whereas it is able to effect the oxidation of *N*,*N*,*N*‘,*N*‘-tetramethyl-p-phenylenediamine (TMPD) with a rate constant of *k* = 1.7 × 10^9^ M^−1^ s^−1^ [29]. Figure 3 also shows the calculated transition state **6** for the water-assisted tautomerization computed at the PCM/B1B95/6-31+G**//B1B95/6-31+G** level. The computed value of 21.3 kJ mol^−1^ is in very good agreement with the experimental value of 23.0 kJ mol^−1^ [11]. Other authors [19] calculated an analogous reaction using the sugar moiety instead of 9-Me at the B3LYP/6-31G(d) level and found an activation barrier of 78.2 kJ mol^−1^, confirming that the choice of theory and the location of the water molecule in the reagents have a profound effect on the outcome. Pulse radiolysis also revealed the “instantaneous” formation of G^•+^ or G(-2H)^•−^ in the reaction of e_aq_^−^ with 8-Br-Guo in acid or basic solutions, and the observed p*K*_a_ values of 3.8 and 10.4 matched well with the reported values of 3.9 and 10.7 for the oxidized Guo, respectively [29].

We discussed above the electron-coupled proton transfer (ECPT) reactions of 8-Br-Guo (**4**) at pH~7. It is also worth mentioning the pulse radiolysis experiments under acidic conditions [29]. At pH 3, the typical spectrum of G^•+^ was generated in two steps: the first one was complete in less than 1 μs and the second one in ~8 μs, corresponding to the reactions of e_aq_^−^ and H^•^, respectively. In the γ-radiolysis study of 8-Br-Guo in aqueous solutions followed by product studies, the formation of Guo as a single product at various pH was shown. In D_2_O solutions, the quantitative incorporation of deuterium at the 8-position was also observed [29].

**Figure 4:**
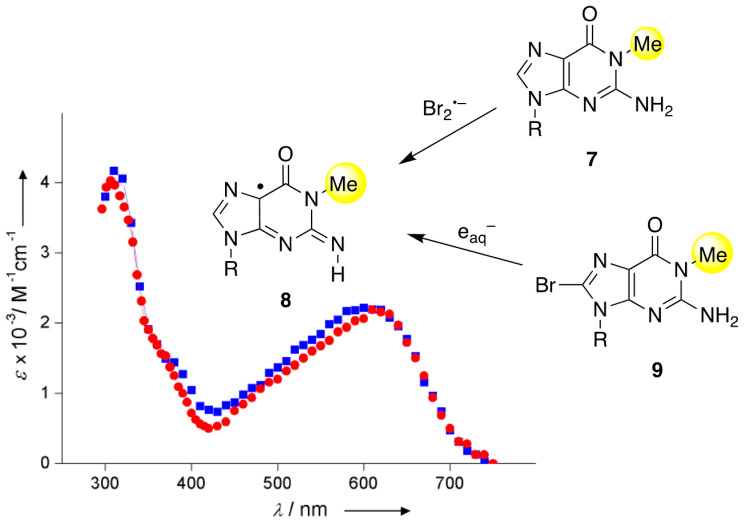
Absorption spectrum (red circles) refers to the oxidation of **7** by Br_2_^•−^ and is assigned to the G(N2-H)^•^ radical (**8**); for the *ε* values, *G*(Br_2_^•−^) = 0.62 μmol J^−1^ was assumed (taken from [14]). The absorption spectrum (blue square) refers to the reduction of **9** by e_aq_^−^ and is assigned to the same G(N2-H)^•^, having presumably the *syn*-conformation; for the *ε* values, *G*(e_aq_^−^) = 0.27 μmol J^−1^ was assumed (taken from [28]). See Appendix A for radiation chemical yields.

A variety of substituted 8-bromoguanine derivatives was also examined. For the tautomerization *syn*-G(N2-H)^•^ ⟶ G(N1-H)^•^ (R = 2′-deoxyribose), the same behavior is observed with an identical rate constant (*k* = 5.0 × 10^4^ s^−1^) when starting from 8-bromo-2′-deoxyguanosine [11]. It is worth mentioning the redox reactions of guanine derivatives having methylation at the N1 position: the oxidation of 1-methyl guanosine (1-Me-Guo, **7**) by Br_2_^•−^ and the reduction of 8-Br-1-Me-Guo (**9**) by e_aq_^−^ showed identical absorption spectra having a band at 610–620 nm, which decays via second-order kinetics (Figure 4). The transient is consistent with the 1-Me-G(N2-H)^•^ radical (**8**) and the absence of tautomerization due to the N1-Me group [11,28]. The ESR and UV-visible spectra of 1-Me-G^•+^ and its corresponding deprotonated 1-Me-G(N2–H)^•^ are also recorded from the one-electron-oxidized 1-Me-dGuo by Cl_2_^•−^ at 77 K at pDs ~5 and ~8 in 7.5 M of LiCl glass/D_2_O, respectively [30]. Identical spectra were observed in the deoxyribose derivative, and the distinction of *syn*- and the *anti*-conformation is not possible experimentally [30].

A thorough comparative computational analysis on the absorption spectra of three guanine-derived radicals (i.e., G^•+^, G(N1-H)^•^, and *syn*-G(N2-H)^•^) in the methyl guanine model was recently reported, indicating that the use of five water molecules of the first solvation shell, the Polarizable Continuum Model (PCM) by TD-DFT based approaches, and the incorporation of vibronic effects provide a good agreement between experiments and theory [31]. The substitution in position 9 of hydrogen in guanosine with a methyl group produces small but noticeable changes in the spectra. 

## 5. Reaction of Hydroxyl Radical with Guanine Derivatives

The reduction potential of HO^•^ is very high (+2.73 V in acid media and +1.90 V at pH 7), but the direct electron-transfer is still rarely observed in HO^•^ reactions [13]. This is also the case for the reaction of HO^•^ with guanine derivatives (**1**), where the redox properties are favorable for electron transfer but H-atom abstraction and addition are observed. 

The reactions of HO^•^ with Guo or dGuo occur with rate constants of ca. 5 × 10^9^ M^−1^ s^−1^. An ambident reactivity has been observed for the reaction of HO^•^ with the G moiety in aqueous medium. In 2000, it was reported that the addition to the base occurs at the C4 position (~65%) to give an adduct, which is the precursor of G(N1-H)^•^ via a dehydration path and at the C8 position (~17%) [32]. The main path (~65%) was based on the oxidizing properties of the transient species (toward TMPD with *k* = ~2 × 10^9^ M^−1^ s^−1^). A few years later, the reaction was revisited by us finding that the main path (~65%) is represented by hydrogen abstraction from the exocyclic NH_2_ group affording G(N2-H)^•^, the oxidizing species that is the precursor of G(N1-H)^•^ via the tautomerization path [27,33]. 

**Figure 5:**
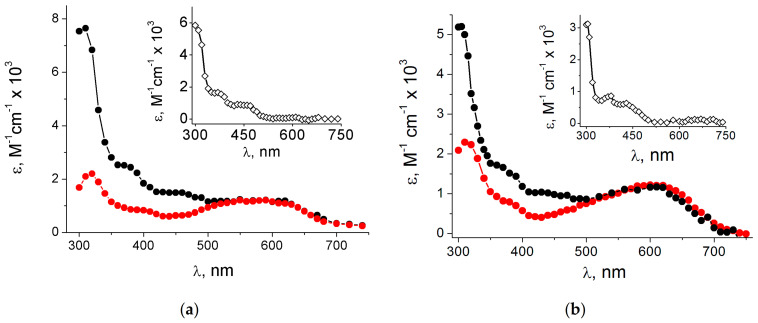
(**a**) Absorption spectra obtained from the reaction of HO^•^ with Guo (**1**) recorded 1 μs (black) after the pulse and 65% of the intensity of the absorption spectra obtained from the reaction of e_aq_^−^ with 8-Br-Guo (**4**) recorded 1 μs (red) after the pulse (inset: the spectrum resulting from the subtraction of red from black) (taken from [27]); (**b**) absorption spectra obtained from the reaction of HO^•^ with 1-Me-Guo (**7**) recorded 1.2 μs (black) after the pulse and 55% of the intensity of the absorption spectra obtained from the reaction of e_aq_^−^ with 8-Br-1-Me-Guo (**9**) recorded 10 μs (red) after the pulse (inset: the spectrum resulting from the subtraction of red from black) (taken from [33]).

**Figure 6:**
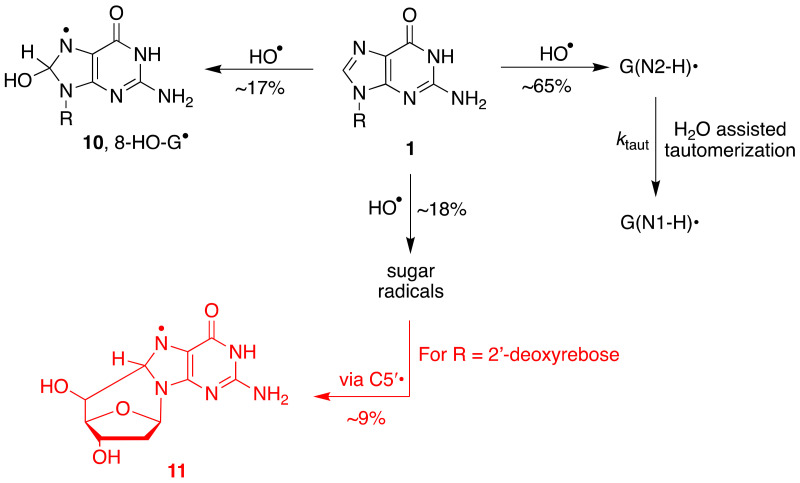
Reaction of HO^•^ with guanine moiety of **1** in aqueous medium. The main path (~65%) is the H-atom abstraction from the exocyclic NH_2_ group to give G(N2-H)^•^ followed by water-assisted tautomerization G(N1-H)^•^. The two minor paths are the addition at the C8 position giving the adduct **10** (~17%) and the hydrogen abstraction from the sugar moiety (~18%). When R = 2′-deoxyrebose, about half of the H-atom abstraction from the sugar occurs at the H5′ positions, followed by cyclization with the formation of radical **11** (adapted from [33]).

The revision was based on the results of a detailed pulse radiolysis with Guo (**1**) and 1-Me-Guo (**7**) and their bromo derivatives 8-Br-Guo (**4**) and 8-Br-1-Me-Guo (**9**) [27,33]. The absorption spectra for the reaction of HO^•^ with **1** and **7** recorded ~1 μs after the pulse are reported in Figure 5a,b (black), respectively. The same figures report the absorption spectra obtained from the reactions of e_aq_^−^ with **4** and **9** (red) described in the previous section. The *ε* values of the black spectra arising from the reaction with HO^•^ radicals were calculated using *G* = 0.61 μmol J^−1^, as both HO^•^ and H^•^ species are scavenged by the guanosine derivatives (cf. Appendix A). On the other hand, both red spectra were calculated using *G* = 0.27 μmol J^−1^ of e_aq_^−^, choosing a percentage of intensity of the corresponding species in order to match the maximum of the spectra around 600 nm. The overlap in the range of 500–700 nm was excellent, choosing 65% and 55% for G(N2-H)^•^ and 1-Me-G(N2-H)^•^, respectively, suggesting that HO^•^ radicals react 65% with Guo (Figure 6) and 55% with 1-Me-Guo by H-atom abstraction from the exocyclic NH_2_ group. The fate of the former is the tautomerization by pseudo-first-order kinetics (*k*_taut_ = 2.3 × 10^4^ s^−1^) to give the tautomer G(N1-H)^•^, whereas the latter follows a second-order decay because the N1 position is blocked. Τhe discussed spectra in the range of 500–700 nm do not contain the contribution from the H-atom reactions, because the H-atom adducts do not absorb at wavelengths above 500 nm [34]. The validation of this analysis is further confirmed when the exocyclic NH_2_ moiety is replaced by NHEt or NEt_2_; the tautomerization G(N2-H)^•^ ⟶ G(N1-H)^•^ occurs only in the first case with a rate constant of 3.6 × 10^4^ s^−1^, whereas, in the second case, a guanyl radical is not formed at all [27,33]. Furthermore, DFT-TD calculations strongly support the structural assignment, as no computed optical transitions were found for the C4-OH adduct of dGuo above 500 nm [27,33]. The insets in Figure 5a,b show the corresponding difference between the two spectra. Both of them show a strong band around 300 nm and various smaller broad bands up to 500 nm. These spectra are expected to be the sum of contributions from the 8-HO and 8-H adducts, as also predicted by the DFT-TD calculations [27,33].

It is also worth mentioning the substitution effect at the C8 position [33]. By replacing the hydrogen of C8–H by an alkyl group, the rate constant of tautomerization is similar (3.0 × 10^4^ s^−1^), whereas a 15-fold increase is observed by replacing H with Br (3.6 × 10^5^ s^−1^). It is worth underlining that the rate constant of tautomerization is found to be 2 times slower than that reported for the reaction of bromo-derivative **4** in Figure 3. The difference in experimental conditions (N_2_O-saturated aqueous solutions vs. Ar-purged aqueous solutions and 0.25 M *t*-BuOH at pH~7) and, more importantly, the overlap of various transient spectra in the reaction of HO^•^ may be the origin of such a difference.

The overall mechanism for the reaction of HO^•^ with Guo and dGuo is illustrated in Figure 6, with the main path (~65%) being the H-atom abstraction from the NH_2_ group and the two minor paths, the formation of the adduct radical **10** (17%) and the H-atom abstraction from the sugar moiety (~18%) [27,33]. A half percentage of this latter path occurs at the H5′ positions and, in the dGuo case, is followed by radical cyclization with the formation of cyclo-derivative **11** [33,35].

It is worth mentioning that time-resolved optical transient absorption spectra for the reaction of HO^•^ with guanine (Gua, free base) was investigated using the pulse radiolysis technique [36]. The reaction was performed at pH 4.6 in order to have mainly the neutral molecule (Gua has three p*K*_a_ values: 3.3, 9.4, and 12.4). The transient absorption spectra were different from those reported for the reaction of HO^•^ with dGuo and Guo, with the 620 nm band particularly being absent. On the basis of the spectral characteristics, reactivity, and quantum chemical calculations, the absorbing species at 330 and 300 nm have been assigned to the C4-OH and C8-OH adducts, respectively [36]. Indeed, a significant difference (more than 200 mV) exists between the oxidation potentials of Gua and Guo or dGuo, where the π electrons play a significant role in the oxidation process, together with the lone electron pair on N9 in the case of the unsubstituted imidazole ring [37]. The reaction of HO^•^ with Gua in an aqueous environment is also addressed by density functional theory (B3LYP) [38]. The calculations suggested the formation of an ion-pair intermediate (G^•+^---OH^–^) that deprotonates to form H_2_O and a neutral G radical, favoring G(N1–H)^•^ over G(N2–H)^•^. The CAM-B3LYP calculation of their UV-visible spectra predicted an absorption around 620 nm [38], which is in contrast with the mentioned experimental results [36]. The theoretical work on the reaction of HO^•^ with Gua [38], often used as a reference for the reaction of the HO^•^ radical with Guo or dGuo or even DNA, is not appropriate. Dizdaroglu in two reviews of 2012 collected and discussed the reactivity of HO^•^ with guanine derivatives without taking into consideration the guanine moiety in different molecular contexts, such as Gua vs. dGuo, and they erroneously questioned the mechanism reported in Figure 6 [39,40].

## 6. Photochemical Precursors

**Figure 7:**
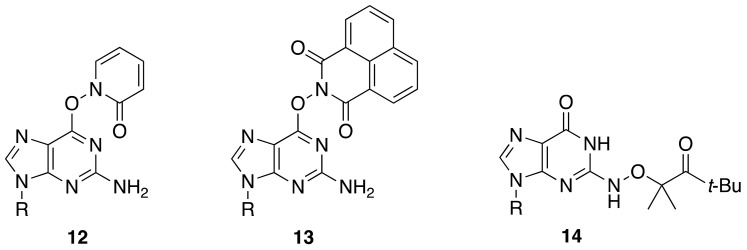
Photochemical precursors **12** and **13** for the formation of G(N1-H)^•^, and **14** for the formation of G(N2-H)^•^.

An alternative approach for guanyl radical generation is based on the photolabile modifier of the guanine moiety. Synthetic precursors **12** and **13** (R = deoxyribose) were designed to generate G(N1-H)^•^ through the homolysis of the N–O bond, and compound **14** was proposed for the selective photo-generation of G(N2-H)^•^ (Figure 7). The G(N1-H)^•^ radical was confirmed for **12** by continuous photolysis product analysis and trapping studies in water, as well as laser flash photolysis experiments [41]. dGuo was formed quantitatively when the precursor **13** is photolyzed under anaerobic conditions in phosphate buffer (pH = 7.2) and in the presence of an excess of β-mercaptoethanol [42]. 

Upon 355 nm laser flash photolysis of **14** (1 mM) and acetophenone (30 mM) in aqueous buffer (pH 7.0)/acetonitrile (1:1, *v*/*v*), the formation of G(N2-H)^•^ is proposed [43]. A broad band between 550 and 700 nm with a maximum at 650 nm was observed, and the authors hypothesized that the broad band contains peaks at 610 and 650 nm belonging to the different conformational isomers of G(N2-H)^•^, in which the remaining N–H bond is either *syn* or *anti* with respect to the guanine N3 atom (cf. Figure 2B). Under their experimental conditions, a variety of possible transients is possible and the observed spectra are an overlap of various species with an over-interpretation regarding the G(N2-H)^•^ radical, which is probably present in a relative low percentage. Indeed, their recorded absorption spectra are in contrast with those reported previously, and no tautomerization was observed within hundreds of microseconds in these experiments. Based on these questionable results, it was suggested that the hydrogen atom abstraction from dG is unlikely to be a major pathway when HO^•^ reacts with dG (see Figure 5 and Figure 6) [43]. Additionally, these authors did not consider that H_2_O-assisted tautomerization depends on the solvent-composition. Water or phosphate buffer (pH 7.0) is not equivalent to the mixture aqueous buffer (pH 7.0)/acetonitrile (1:1, *v*/*v*). The microheterogeneities of water–acetonitrile mixtures are well documented (see e.g., refs [44,45,46]). The independent generation of the G(N2-H)^•^ radical by photochemical precursors for time-resolved spectroscopic studies needs to be addressed by further research. 

## 7. The Fate of Guanyl Radical in Nucleosides

As shown above, guanine can be oxidized to G^•+^ by a variety of oxidants such as SO_4_^•−^, Br_2_^•−^, Cl_2_^•−^, and CO_3_^•−^, including various metal complexes. The carbonate radical anion (CO_3_^•−^) is an important reactive species under physiological conditions. The pair CO_2_/HCO_3_^−^ is an active buffer maintaining physiological pH, and HCO_3_^−^ exists in millimolar levels in vivo. The reaction of CO_2_ with peroxynitrite (ONOO^−^) generates CO_3_^•−^ through the unstable nitrosoperoxycarbonate [47]. The reaction of HO^•^ with HCO_3_^−^ produces CO_3_^•−^. The Fenton reaction in the presence of HCO_3_^−^ affords CO_3_^•−^ rather than the HO^•^ radical [48]. The reduction potential of CO_3_^•−^/CO_3_ is 1.59 V; therefore, CO_3_^•−^ is a milder single-electron oxidant that abstracts an electron from guanine moieties [49]. The oxidation mechanism and kinetics of dGuo (**1**) by the carbonate radical anion (CO_3_^•−^) have also been investigated theoretically [50]. 

**Figure 8:**
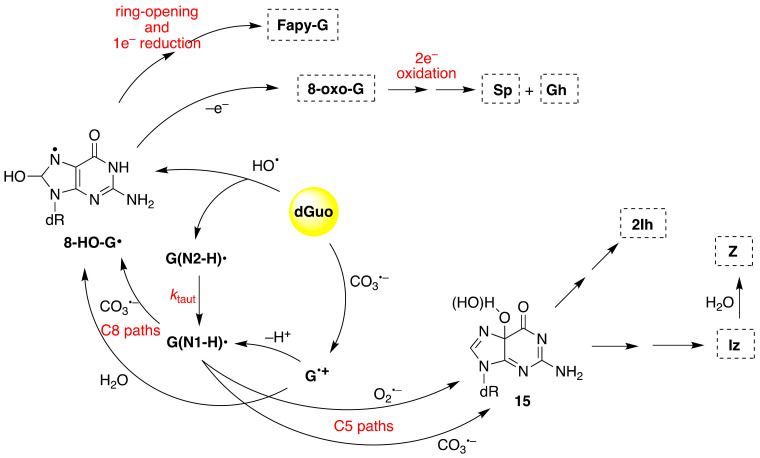
The mechanism of dGuo (**1**) oxidation by hydroxyl radical (HO^•^) or carbonate radical anion (CO_3_^•−^); for the chemical structures of 8-oxo-G, Fapy-G, Sp, Gh, 2Ih, Iz, and Z, see Figure 15.

The iron-induced Fenton oxidation of dGuo (**1**) in the presence of bicarbonate buffer and in its absence (phosphate buffer) affords different distribution products consistent with CO_3_^•−^ and HO^•^ reactive intermediates, respectively [51]. Figure 8 shows the overall mechanism of oxidation of dGuo by HO^•^ and CO_3_^•−^. The two oxidants have some common intermediates generated by different routes, e.g., (N1-H)^•^ and 8-HO-G^•^ radicals. The 8-HO-G^•^ is the common precursor of 8-oxo-G and Fapy-G. The Fapy-G formation requires ring-opening followed by one-electron reduction or vice versa (i.e., one-electron reduction followed by ring-opening). The formation of Fapy-G depends on the oxygen concentration and the redox environment [52,53]. 8-oxo-G, with a reduction potential 0.55 V lower than that of G [54], undergoes further oxidation in the presence of one-electron oxidants. Two main products derive from the oxidation of 8-oxo-G, namely spiroiminodihydantoin (Sp) and 5-guanidinohydantoin-2′-deoxyribose (Gh) [55]. From the oxidation of dGuo under aerobic conditions or in the presence of CO_3_^•–^, two other products are formed via C5 paths (Figure 8), viz. 5-carboxamido-5-formamido-2- iminohydantoin-2′-deoxyribonucleoside (2Ih) and 2-amino-5-[2-deoxyribose]-4H-imidazol-4-one (Iz). The latter is further hydrolyzed to 2,2-diamino-4-[(2-deoxyribose)amino]-5(2H)-oxazolone (Z) [56,57]. 

## 8. Guanyl Radicals in ss-ODNs and ds-ODNs

The mode of formation and the kinetics of decay of G^•+^ depend strongly on ODN secondary structures. In this section, we consider ss- and ds-ODNs, whereas the ODNs arranged in G-quadruplexes are reported in the next section. 

**Figure 9:**
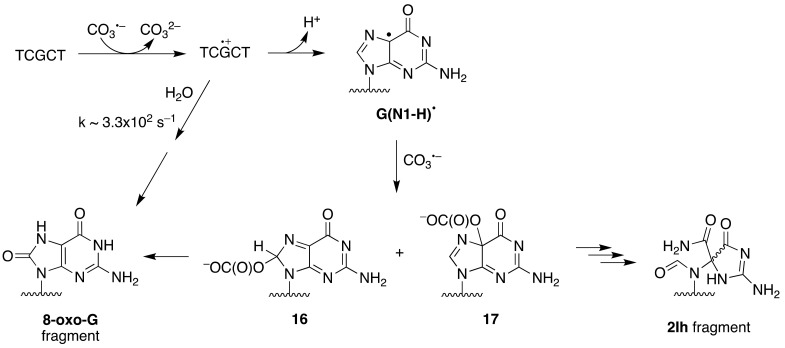
Mechanistic scheme of one-electron oxidation of ss-ODN (TCGCT) by carbonate radical anion CO_3_^•−^ (adapted from ref. [54].

The one-electron oxidation of ss-ODN (TCGCT) by CO_3_^•−^ and SO_4_^•−^ was studied by laser flash photolysis in some detail [58]. Both reactions lead to the formation of the guanine radical cation G^•+^ and/or its deprotonation product G(N1-H)^•^ depending on pH (p*K*_a_ = 3.9) (Figure 9). At pH 2.5, the G^•+^ radical is hydrated within 3 ms, forming 8-oxo-dG in the ODN. At pH 7.0, G(N1-H)^•^ reacts only slowly with H_2_O and lives for ∼70 ms, ultimately giving intrastrand cross-link adducts. Alternatively, it can be further oxidized by reaction with CO_3_^•−^, generating the two-electron oxidation products 8-oxo-dG (via C8 addition **16**) and 2Ih (via C5 addition **17**). The same group has studied the one-electron oxidation of a 30mer ss-ODN containing eleven Gs in comparison with the same strand in ds-ODN, and these results are discussed below. 

Since early works, it has been postulated that in DNA, the proton is not directly lost from G^•+^ to the aqueous phase but remains within the hydrogen-bonded G^•+^:::C pair located toward the cytosine N3 atom [12]. ESR studies of the one-electron oxidation of a variety of ds-ODNs indicated that the proton is entirely transferred [G(N1-H)^•^:::CH^+^] at 77 K [59], whereas a prototropic equilibrium [G^•+^:::C ⇆ G(N1-H)^•^:::CH^+^] should be established at ambient temperature. Most of the experimental studies described below on ds-ODNs used SO_4_^•−^ as the oxidizing agent. It should be recalled that SO_4_^•−^ reacts with all four nucleobases (G, C, A, and T) with rate constants that are close to diffusion-controlled rates [13], although the primary damage is localized at Gs, having the lowest reduction potential (cf. Section 1) [4,5,6,7,8,9,10].

**Figure 10:**
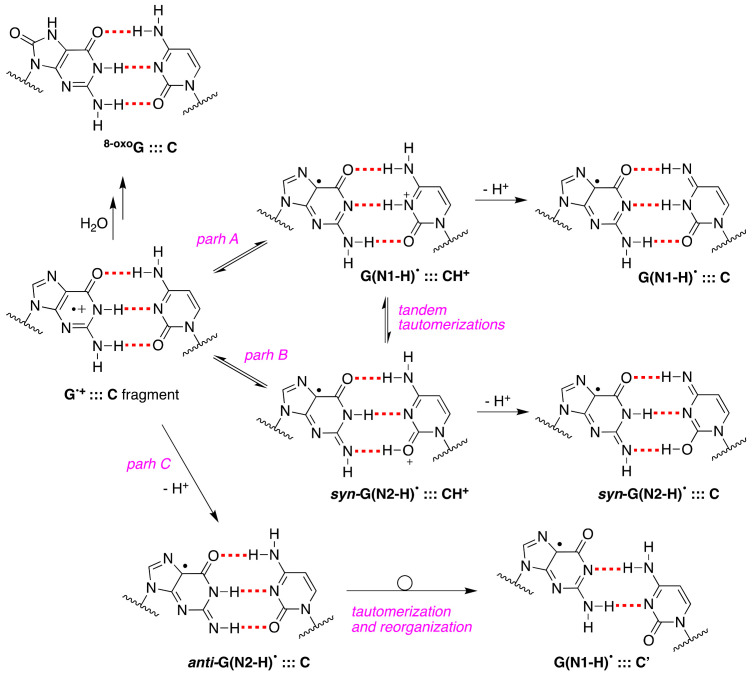
Mechanistic possibilities of G^•+^:::C deprotonation in ds-ODNs and its reaction with water to give ^8-oxo^G:::C.

The one-electron oxidation by the powerful SO_4_^•–^ of a variety of ds-ODNs containing G:::C pairs has been reported in pulse radiolysis [16,60] or laser flash photolysis [61] studies. Pulse radiolysis with optical detection showed that different ds-ODNs containing G, GG, and GGG sequences afford the corresponding radical cation G^•+^:::C. In the earlier work [16], a biphasic decay of G^+•^:::C was assigned to the shift in the N1 proton in G^+•^ to its partner C, followed by the release of the proton into the solution. The same group revisited these reactions by analyzing the optical spectra and the kinetics of eleven ds-ODNs with different sequences (11mer to 13mer ds-ODNs) [60]. The revised results concern the monophasic decay of [G^•+^:::C ⇆ G(N1-H)^•^:::CH^+^] associated with the release of the proton into solution. Based on p*K*_a_ values, the population of positive charges is estimated to be ~1:3 in favor of cytosine [12]. The rate constant for deprotonation was dependent on the ds-ODN sequence, especially on the sequence of bases adjacent to the guanine base, varying in the range of 0.3–2 × 10^7^ s^−1^ by monitoring the transient. In the ds-ODN of the 13mer A_5_G_3_A_5_, the first transient decays with *k* = 4.5 × 10^6^ s^−1^ in H_2_O, showing a *k*(H_2_O)/*k*(D_2_O) of 3.8-fold. Interestingly, by replacing C with 5-Me-C or 5-Br-C in ds-ODN, the rate constant of deprotonation increased or decreased, respectively. Overall, the proposed mechanism consists of path A in Figure 10 [60].

Upon our discovery of the first directly observed differences of the two tautomeric forms of one-electron-oxidized Guo or dGuo (see Figure 3), we proposed that an alternative scenario of G^•+^:::C deprotonation involving the tautomer G(N2-H)^•^ should also be considered in future studies; in particular, using time-resolved studies, the monitoring of the absorbance changes around 620 nm is important, as G(N2-H)^•^ has a distinct absorption [11]. Figure 10 shows the proposed paths B and C that involve *syn*-G(N2-H)^•^ and *anti*-G(N2-H)^•^, respectively (cf. Figure 2B). In path C, the protons can escape directly into the bulk solution forming *anti*-G(N2-H)^•^, whereas, in path B, a proton-transfer equilibrium involves the *syn*-G(N2-H)^•^ conformer that is calculated to be more stable. We were occasionally criticized for such a proposal, but, today, it is gratifying to see that such a “second face” of the guanyl radical, i.e., G(N2-H)^•^, is indeed recalled as an intermediate in several oxidation reactions of secondary structures of DNA (vide infra). 

For the first time, the presence of *ant*i-G(N2–H)^•^:::C in a ds-ODN is shown to be easily distinguished from the other prototropic forms, owing to its readily observable nitrogen hyperfine coupling by electron spin resonance (ESR) [30]. Indeed, the one-oxidation of d[TGCGCGCA]_2_ has been studied in detail by ESR and UV-visible spectral analysis; at pH ≥ 7, the initial site of deprotonation is found to be at N1, forming G(N1–H)^•^:::C at 155 K, and upon annealing to 175 K, the site of deprotonation to the solvent shifts to an equilibrium mixture of G(N1–H)^•^:::C and *anti*-G(N2–H)^•^:::C. This ESR identification is supported by the visible absorption at 630 nm, which is characteristic for G(N2–H)^•^ radicals. 

**Figure 11:**
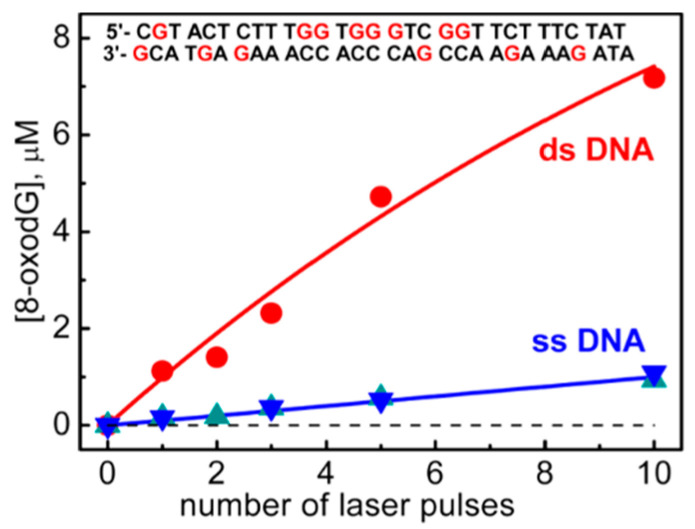
Dependence of the yields of 8-oxodGuo lesions in ds-ODN vs. ss-ODN on the number of successive 308 nm laser pulses. Taken from [58].

The one-electron oxidation of 30mer ODNs by SO_4_^•−^, shown in Figure 11, was studied in ss-ODNs or ds-ODN by time-resolved absorption spectroscopy and quantification of the 8-oxo-G lesion as the final product [61]. Figure 11 shows the dependence of the yields of 8-oxo-dG in ds-ODN vs. ss-ODNs on the number of successive 308 nm laser pulses. The yield of 8-oxo-G in ds-ODN is ~7 times higher than that in ss-ODNs, and the initial 8-oxo-G yields are ~1 μM per laser pulse that corresponds to ~20% per G(-H)^•^ radical generated. Therefore, the secondary structure of ds-ODN is a crucial factor that enhances the formation of 8-oxo-G lesions from G(N1-H)^•^ radicals. The transient species observed after the complete decay of SO_4_^•–^ (5 μs) for both ds-ODN and ss-ODN were very similar and assigned to G(N1-H)^•^. However, the kinetics of G(N1-H)^•^ decay were quite different; in ds-ODN, the decay is biphasic with one component decaying with a lifetime of ~2.2 ms and the other one with a lifetime of ~0.18 s, whereas, in ss-ODN, the decay is monophasic with a ~0.28 s lifetime. The millisecond decay component in ds-ODN is correlated with the enhancement of 8-oxo-G yields, which are ~7 times greater than those in ss-ODN. In ds-ODN, the authors proposed that the equilibrium [G^•+^:::C ⇆ G(N1-H)^•^:::CH^+^] allows for the hydration followed by the formation of ^8-oxo^G:::C (see Figure 10). By contrast, in ss-ODN, the deprotonation of G^•+^ and the irreversible escape of the proton into the aqueous phase compete more effectively with the hydration mechanism, thus diminishing the yield of 8-oxo-G. In order to accommodate the fast deprotonation (see above) [60], they invoked path C in Figure 10, followed by a tautomerization of G(N2-H)^•^ to G(N1-H)^•^ coupled with base displacement and reorganization of the hydrogen-bonding network [61]. 

It was demonstrated that absorption of a single low-energy UV photon by ds-ODNs at 265 nm generates guanyl radicals, with an appreciable quantum yield (>10^−3^). The transient species detected after 3 μs are identified as guanyl radicals G(N1-H)^•^, which decay with a half-life of 2.5 ms. The experimental results corroborated by theoretical studies suggest nonvertical processes, associated with the relaxation of electronic excited states [62,63,64]. Similar results were obtained with ct-DNA [64]. The same ss-ODNs and ds-ODN reported in Figure 11 were studied by photoionization using ultra-purified chemicals [63]. Although the spectra on the microsecond timescale are the same as those reported [61], the radical lifetimes were found to be much shorter. It was also shown that base pairing slows down the radical lifetime, and this was attributed to the fact that larger conformational motions in single strands allow the systems to adopt more rapidly reactive conformations [63].

It is also worth citing the pulse radiolysis studies using the selenite radical, SeO_3_^•−^ of *E*^0^ 1.68 V, oxidizing DNA with a rate constant of 3.5 × 10^7^ M^−1^ s^−1^ [65]. The one-electron oxidation of ct-DNA by SeO_3_^•−^ involves the G:::C pair in a different way, as it was proposed via a concerted formation of a cytosyl radical followed by proton loss from the amine substituent of cytosine into the major groove and the H-atom transfer to the guanyl radical G(N1-H)^•^:::C [65]. The neutral radical, *anti*-G(N2-H)^•^:::C, in DNA was formed directly through H-atom abstraction from its amine moiety by the benzotriazinyl radical, and a DFT calculation corroborated such a pathway; the *E*_7_ value of 1.22 V for *anti*-G(N2-H)^•^:::C has been determined by analysis of both absorption and kinetic data [66]. Moreover, an investigation of the aniline radical cation, (ArNH_2_)^•+^, with ds-ODN or DNA suggested the formation of the guanyl radical in an energetically more stable ”slipped” structure, similar to the one reported in Figure 10 (bottom-right) [67].

Much attention has been focused on computing the properties of the G^•+^:::C pair fragment of DNA (e.g., the proton transfer equilibria, hydrated cation, tautomerism or redox behavior) by DFT calculations including solvent effects. The theoretical calculations have been abundant during the last two decades in this area, and it is not the scope of this review to examine the whole scenario of the reported calculations. Only a few with more attention on recent publications are reported; in particular, the influence of hydration on the proton transfer G^•+^:::C pair [68,69,70]; the calculations for a single GC base pair, suggesting a very slight favor of *anti*-G(N2–H)^•^:::C by 1.7 kJ mol^–1^ over the G(N1–H)^•^:::C structure [30]; and the calculations providing reference absorption spectra for guanine radicals in duplexes and the computed spectra that predict changes in transient absorption spectra expected for hole localization, as well as for deprotonation (to cytosine and bulk water) and hydration of the radical cation [62,71]. 

It is worth mentioning that oxyl radicals such as O_2_^•−^, NO_2_^•^, and CO_3_^•−^ react with G(N1-H)^•^ of ss-ODNs or ds-ODNs in cross-radical termination mode and, in all cases, the combination occurs via the analogous C8 and C5 paths reported in Figure 8 with the formation of expected end-products [72,73,74]. The ratio of the C5/C8 products can depend on the macromolecular secondary structure, e.g., in the case of NO_2_^•^, the ratio of C5/C8 addition decreases from 2.1−2.6 in ss-ODNs to 0.8−1.1 in ds-ODNs [74]. The influence of the local base sequence and secondary structure on G oxidation by riboflavin-mediated photooxidation, and nitrosoperoxycarbonate with the formation of 8-oxo-G, Z, and 8-NO_2_-G lesions are also reported [75]. 

The radical chemistry reported for Guo or dGuo is often transferred to complex macromolecular arrangements such as the secondary DNA structure. From this section, it is clear that the one-electron oxidation of ds-ODNs or DNA is a complex matter with many variants, including chemical methods of generation, time-resolved spectroscopic approaches, base sequences, and thermochemical cascade. Mechanistic proposals are based on multidisciplinary knowledge, where DFT calculations can often help for a better understanding of the favored pathways but must be combined with the experimental evidence.

## 9. Guanyl Radicals in G-Quadruplex

**Figure 12:**
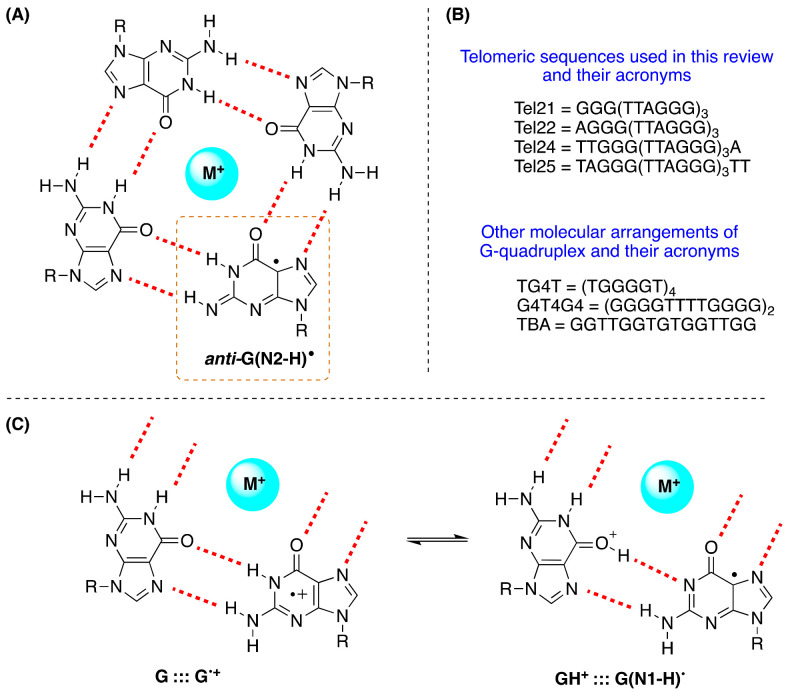
(**A**) Drawing of G-quartet containing the guanyl radical *anti*-G(N2-H)^•^ (cf. Figure 2B); (**B**) the ss-ODNs sequences used in this review (Section 9 and Section 10) in G-quadruplex structures; (**C**) fragments of G-quartet; the proposed equilibrium in one-electron-oxidized G quartet [78].

The structure of the G-quadruplex consists of stacked G-quartets where each G-quartet is a planar array of four Hoogsteen-bonded guanines stabilized by metal cations [76]. The existence of G^•+^ in the G-quadruplex and their deprotonation from the NH_2_ moiety of guanines was first reported in a study performed via photosensitization (Figure 12A) [77]. The four G-quadruplexes buffered using Na^+^ or K^+^, i.e., Tel22/Na^+^, TG4T/K^+^, G4T4G4/Na^+^, and the TBA/K^+^, are used, that adopt three of them antiparallel and TG4T/K^+^ parallel orientations (see Figure 12B). The one-electron oxidation was carried out by SO_4_^•−^ using laser flash photolysis for the transient detection. The deprotonation rate of G^•+^ within G-quadruplexes Tel22/Na^+^, TG4T/K^+^, and G4T4G4/Na^+^ (*k* = ~2 × 10^5^ s^−1^) was found to be 1−2 orders of magnitude slower compared to G^•+^ from dGuo or ds-ODNs with the formation of the G(N2-H)^•^ radical in *anti*-conformation (Figure 12A), whereas in TBA/K^+^, the deprotonation occurred from the G in the loop affording G(N1−H)^•^ with a rate constant of 1.4 × 10^6^ s^−1^ [77]. 

In antithesis with the above-mentioned work [77], the one-electron oxidation of Tel25, buffered using Na^+^ or K^+^, by SO_4_^•−^ was also investigated by time-resolved spectroscopy and determined the end-product 8-oxo-G. In neutral aqueous solutions (pH 7.0), the observed transient is G(N1-H)^•^ after the complete decay of SO_4_^•−^ (~10 μs after the actinic laser flash). In both systems, the G(-H)^•^ decay is biphasic with one component decaying with a lifetime of ~0.1 ms, and the other one with a lifetime of 20–30 ms. The fast decay component (~0.1 ms) in G-quadruplexes is correlated with the formation of 8-oxo-G lesions. The authors proposed that in G-quadruplexes, G(-H)^•^ radicals retain their radical cation character by sharing the N1-proton with the O6-atom of G in the [G^•+^:::G] Hoogsteen base pair (Figure 12C); this equilibrium [G:::G^•+^ ⇆ GH^+^:::G(N1-H)^•^] leads to the hydration of G^•+^ within the millisecond time domain, and is followed by the formation of the 8-oxo-G lesions [78].

The first study on the UV-induced ionization of G-quadruplexes was reported by Markovitsi and co-workers [79]. Using nanosecond time-resolved spectroscopy corroborated by TD-DFT calculations and the telomeric sequence Tel21 (cf. Figure 12B) in buffered solutions containing Na^+^ cations, they showed that irradiation at 266 nm, corresponding to an energy significantly lower than the guanine ionization potential, provokes one-photon ionization in aqueous solution, affording the guanyl radical. The fate of guanine radicals is followed over five orders of magnitude of time: (i) up to 30 ns 100% G^•+^, (ii) in 3 μs 50% G^•+^ and 50% *ant*i-G(N2-H)^•^ that become 35% G^•+^ and 50% G(N2-H)^•^ in 20 μs; (iii) in 5 ms 50% G(N1-H)^•^ that reaches 6% in 180 ms. The two main features are a long-lived G^•+^ with respect to ds-ODNs due to the secondary structure and the detection of the two guanyl radicals, including the tautomerization *anti*-G(N2-H)^•^ → G(N1-H)^•^, which takes place on the millisecond timescale. The calculations indicated that *anti*-G(N2-H)^•^ is 12.1 kJ mol^−1^ more stable than the G(N1-H)^•^ in the simulated environment of Tel21/Na^+^ [79]. Similar behaviors were also reported for Tel25/Na^+^ [63] and TG4T/Na^+^ [80], although their guanines adopt, respectively, antiparallel and parallel orientations in respect of the glycosidic bonds. By replacing Na^+^ with K^+^ in Tel21 [81] and TG4T [82], some important changes were observed: the quantum yield of one-photon ionization at 266 nm was twice as high in both systems and the *ant*i-G(N2-H)^•^ radical decayed faster. Moreover, in the case of TG4T/K^+^, the tautomerization step G(N2-H)^•^ → G(N1-H)^•^ is suppressed. Such a behavior shows that the nature of the metal ion plays an important role in the reaction mechanism.

It is also worth mentioning the formation of 8-oxo-G in the comparative study on Tel21/Na^+^ and TG4T/Na^+^, where for the former, the yield of 8-oxo-G is much higher than that of the latter [79,80]. As the fate of G^•+^ is split between hydration vs. deprotonation, the authors suggested that the G^•+^ population survives on the millisecond timescale in Tel21/Na^+^, whereas the deprotonation is much faster in TG4T/Na^+^, thus rendering the reaction path leading to 8-oxodG less probable. Markovitsi and co-workers recently summarized their work on the guanyl radicals in ds-ODNs and G-quadruplexes [64,83]. 

Two main questions remain open on the guanyl radical in G-quadruplexes: (i) what is the mechanism of tautomerization *ant*i-G(N2-H)^•^ → G(N1-H)^•^, and (ii) based on the results described above, the major oxidative damage is expected to arise from deprotonated radicals; what are the end-products of the two guanyl radicals and their relation with the 8-oxo-G lesion. Theoretical calculations combined with time-resolved methodologies and product studies will certainly help understand such pending radical mechanisms.

## 10. Reaction of Hydroxyl Radical with ODNs and DNA

The reaction of HO^•^ with G in the macromolecular environment is worth a special comment, because the behavior is quite different from the reaction of HO^•^ with simple Guo or dGuo (Section 5). The four common nucleosides of DNA (dGuo, dAdo, dCyt, and Thy) react with HO^•^ at close to diffusion-controlled rates (4–7 × 10^9^ M^−1^ s^−1^), with the pyrimidine nucleosides being slightly higher [13]. The sites of DNA attack by diffusible HO^•^ radicals are known to be either the hydrogen atom abstraction from the 2′-deoxyribose units or the addition to the base moieties, with the latter being the predominant one (accounting for 85–90% of attacked sites) [13].

**Figure 13:**
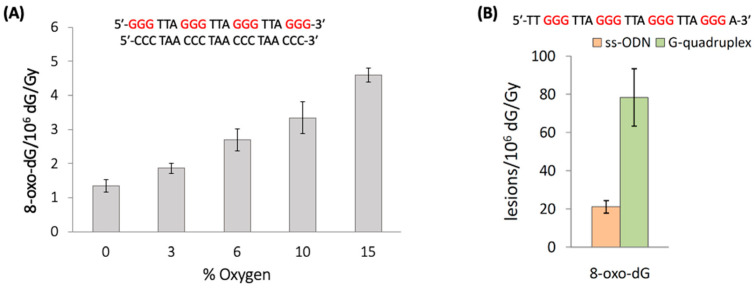
(**A**) Radiation-induced formation of 8-oxo-dG in ds-ODNs at increasing concentrations of oxygen; data from [84]. (**B**) Radiation-induced formation of 8-oxo-dG lesions in ss-ODN (nonfolded state) and G-quadruplex (folded state) in deareated conditions; the G-quadruplex was prepared by dissolving the oligonucleotides in 10 mM of KH_2_PO_4_ buffer containing 70 mM of KClO_4_ (pH 7.0). Values represent the mean ± SD per 10^6^ dG per Gy of γ-irradiation in the range from 0 to 60 Gy of irradiation.

**Figure 14:**
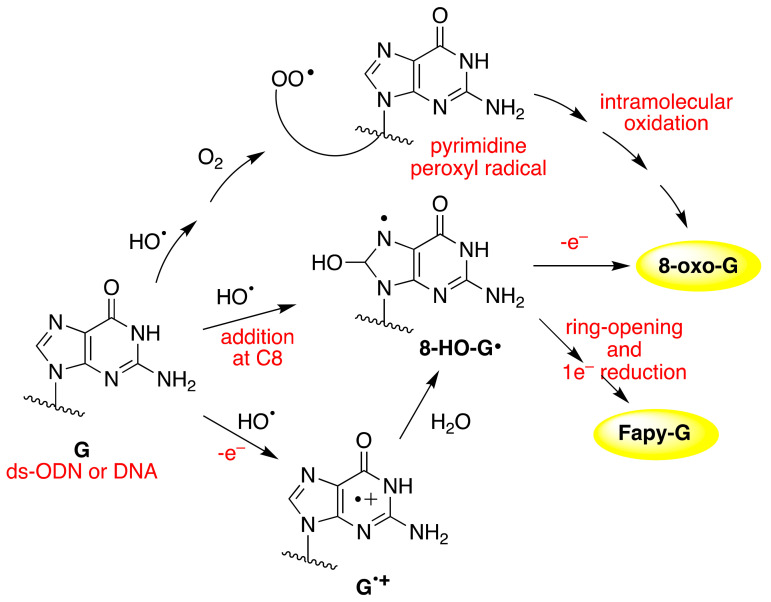
Reaction mechanism for the formation of 8-oxo-G in ds-ODN. For the hydration reaction of G^•+^ and identification of 8-HO-G^•^ by electron spin resonance (ESR) in DNA, see reference [89].

The reaction of HO^•^ with the 21-mer ds-ODNs reported in Figure 13A shows that increasing concentrations of oxygen lead to elevated levels of 8-oxo-G. The 8-oxo-G levels vary from 1.35 8-oxo-dG/10^6^ dG/Gy in the absence of O_2_ up to 4.60 for 2.01 × 10^−4^ M of O_2_ (15%) [84]. The mechanism of the formation of 8-oxo-G by the reaction of HO^•^ with ct-DNA and ds-ODNs in the presence of oxygen has been investigated in detail [85,86,87]. It was demonstrated that three pathways contribute to the formation of 8-oxo-dG, as shown in Figure 14. The direct addition of HO^•^ to the C8 position of the guanine moiety to form 8-HO-G^•^ accounts for the minor path (~5%), whereas the remaining ~95% of 8-oxo-dG are produced by two types of reactions in approximately equal amounts involving DNA radicals, that is, one-electron oxidation (probably by a variety of reactions not very well understood) followed by hydration reaction (~45%) or an intramolecular addition of a transiently generated pyrimidine peroxyl radical onto the C8 of a vicinal guanine base (~50%). As mentioned earlier, the Fapy-G lesions are chemically related to 8-oxo-G as they are generated from the same intermediate 8-HO-dG^•^ (Figure 14). The formation of Fapy-G is dependent on the oxygen concentration and the redox environment. [52,53].

A pulse radiolysis study reported the reaction of four 12mer ONDs with four or two Gs subjected, either as ss-ODNs or ds-ODNs, to the reaction with HO^•^ radicals using an intensified charge-coupled device (ICCD) as an alternative to the photomultiplier tube (PMT) for transient spectra measurements [88]. The characteristic band >600 nm of the guanyl radical G(N2-H)^•^ was present in ss-ODNs and disappeared on the microsecond timescale, but it was absent in ds-ODNs. 

The impact of G-quadruplex folding with respect to unfolded sequences in ss-ODNs on the formation of 8-oxo-G lesions was also investigated [90]. In particular, the unfolded sequences of TG4T and mutated Tel24, as well as the G-quadruplexes of TG4T/K^+^ and Tel24/K^+^ (see Figure 12B), were exposed to HO^•^ radicals generated by γ-radiolysis, and the 8-oxo-G was then quantified via stable isotope dilution LC-MS/MS analysis. Figure 13B shows the results of Tel24, where 8-oxo-G lesions formation in the folded G-quadruplex is ~4 folds higher than the unfolded sequences (78.4 vs. 21.2 of 8-oxo-G/10^6^ dG/Gy, respectively). In the TG4T system, the 8-oxo-G lesions formation in tetramolecular parallel-stranded G-quadruplex is ~2.8 folds higher than the monomolecular sequences (39.9 vs. 14.4 of 8-oxo-G/10^6^ dG/Gy, respectively). Therefore, the self-organization of guanines in the G-quadruplex tetrads produced ~3- and ~4-fold increases in 8-oxo-G yields, indicating that the G-quadruplex is significantly more susceptible to HO^•^ oxidation. The mechanism of the formation of 8-oxo-G by HO^•^ in G-quadruplexes is not clear at present, with the addition at the C8 position being the most reasonable key step.

## 11. Biomarkers of Guanine DNA Damage

Reactive oxygen and nitrogen species (ROS/RNS) are produced in a wide range of physiological processes and are also responsible for a variety of pathological processes [1,2,3]. Persistent oxidative stress developed at sites of chronic inflammation is characterized by overproduction of ROS/RNS, which targets cellular DNA, and, as a consequence, may lead to mutations and cancer. For example, the 8-oxo-G lesion is genotoxic [91], and failure to remove this lesion before replication induces the G:::C → T:::A transversion mutation.

**Figure 15:**
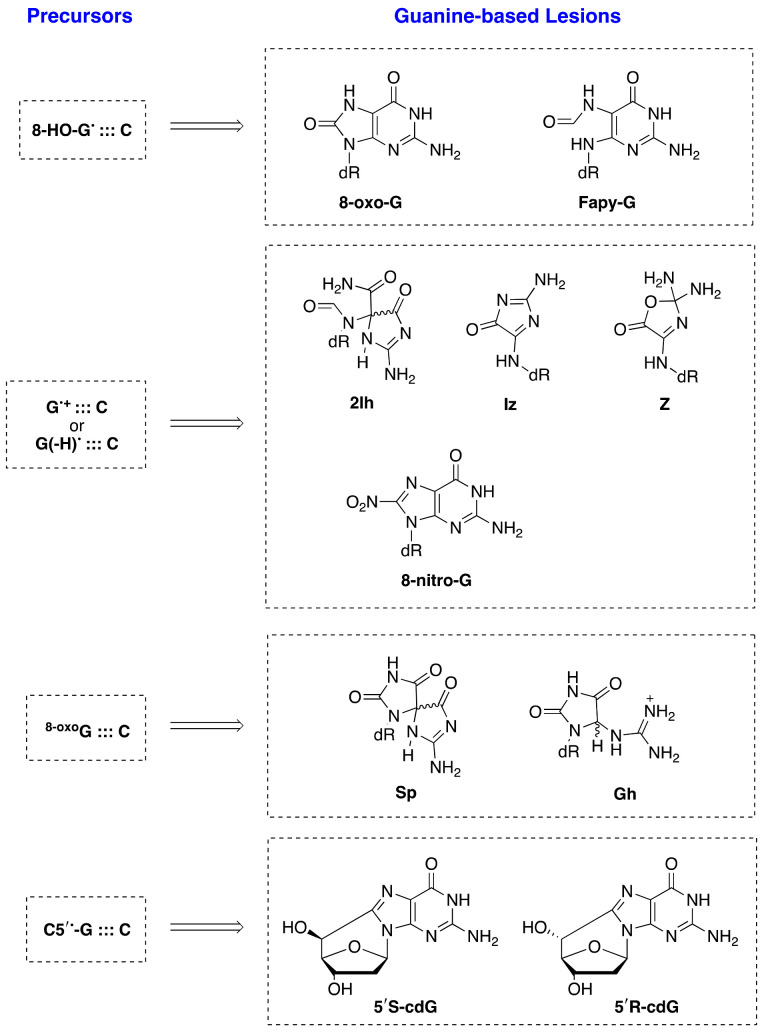
(**Left**) The precursor of the lesions in ds-ODNs or DNA. (**Right**) Guanine-derived lesions detected in in vitro experiments by the reaction of reactive oxygen species (ROS) with ds-ODNs or naked DNA. Most of these lesions have also been found after oxidatively induced DNA damage in vivo.

The ROS/RNS network includes molecules such as hydrogen peroxide (H_2_O_2_), hypochlorous acid (HOCl), and peroxynitrite (ONOO^−^), as well as radicals such as the superoxide radical anion (O_2_^•−^), nitric oxide (NO^•^), hydroxyl radical (HO^•^), nitrogen dioxide (NO_2_^•^), and carbonate radical anion (CO_3_^•−^). Most of these species can react with DNA, and the nucleobase guanine with the lowest reduction potential is the dominant site for oxidation within DNA through the hole transfer [4,5,6,7,8,9,10]. Analytical protocols and, in particular, liquid chromatography–tandem mass spectrometry (LC-MS/MS) allow the identification of the DNA lesions with high-accuracy in cellular DNA after enzymatic digestion, as reviewed recently [92,93]. The main guanine-derived lesions observed in vitro and/or in vivo from oxidatively generated DNA damage are collected in Figure 15 and divided in four groups, depending on the precursor. In model DNA studies, the relative yields of these lesions depend on the reaction context, and several comparative studies with various types of oxidants are reported [94]. 

8-Oxo-G and Fapy-G are chemically connected because they derive from the same radical precursor (8-hydroxyl radical adduct, 8-HO-G^•^) and their comparisons have been extensively treated [52,92]. Fapy-G due to its instability is mainly detected as the free base modification (i.e., Fapy-Gua), and both GC-MS/MS and LC-MS/MS techniques with the isotope dilution approach have been used for the measurements in vitro and in vivo [95]. A large number of publications deal with these lesions not only in vitro but also in vivo [96,97]. There are a few studies at the macromolecular level in vitro for the formation of Ih, Iz, Z, Sp, and Gh lesions. The Sp and Gh lesions derive from further oxidation products of 8-oxo-G, whereas the d2Ih, dIz, and dZ lesions are the final products of the further reaction of G^•+^ or G(N1-H)^•^ with oxyl radicals generated under aerobic conditions in vitro. Comparative analyses of the four oxidized G lesions (8-oxo-G, Z, Sp, and Gh) from the reaction of ct-DNA with HO^•^, ONOO^–^, and ^1^O_2_ [98], as well as of four oxidized G lesions (8-oxo-G, Z, and 8-NO_2_-G) from the reaction of ds-ODNs with HO^•^ and NO_2_^•^/CO_3_^•−^ [75], were examined using LC–MS/MS and isotopomeric internal standards. There are a few studies in murine models reporting the detection of dSp, dGh, and dZ [99].

5′,8-Cyclo-2′-deoxyguanosine (cdG) in its 5′R and 5′S diastereoisomeric forms results from the C5′ radical cyclization on the C8 position of G (see Figure 5) [92,93]. Although the guanine DNA lesions mentioned above are generated by various ROS (including HO^•^), the formation of 5′R-cdG and 5′S-cdG lesions in vitro and in vivo relies exclusively on the HO^•^ attack. Recent results on the quantification of 5′R-cdG, 5′S-cdG, and 8-oxo-G lesions in various types of biological specimens associated with the cellular repair efficiency, as well as with distinct pathologies, have been reported, providing some insights on their biological significance [92].

Base excision repair (BER) is the major enzymatic pathway involved in the processing of base damages such as 8-oxo-G and Fapy-G by creating an abasic site [100]. Nucleotide excision repair (NER) pathways are the major cellular pathway for the repair of bulky adducts and other helix-distorting lesions such as 5′R-cdG and 5′S-cdG lesions [93]. Evidence has also been provided that the removal of some oxidatively generated purine DNA lesions involves the overlap of both pathways [101,102].

## 12. Conclusions

The one-electron oxidation of the guanine moiety (G^•+^) is the most relevant process generating transient precursors involved in the oxidative damage of DNA with a variety of end-products, including the 8-oxo-dG lesion. There is a plethora of experimental and theoretical papers dealing with the well-understood long-range charge transport in DNA with the final destination of the G sites. Upon formation of G^•+^, two main paths are recognized: (i) a nucleophilic attack, such as the hydration reaction with the formation of 8-HOG^•^, and (ii) the prototropic equilibrium [G^•+^:::C ⇆ G(-H)^•^:::CH^+^]. Both are at the crossroads of various pathways to the end-products. Furthermore, the one-electron oxidation of the G-quadruplex, with their planar array stabilized by metal cations, represent another context of G^•+^ fate.

For such a complex chemistry, it is obvious that the single nucleosides such as Guo or dGuo and the free base Gua have been the prototypes for experiments and theoretical calculations. However, neither Gua represents dGuo nor dGuo represents ODNs in their various supramolecular arrangements, and the extrapolation of its free radical chemistry can generate confusion. For example, for the introduction of the HO^•^ radical reactivity toward DNA, the main path reported is the addition at the C4 position followed by dehydration. There is no evidence for the existence of such a reaction. Overall, the reaction of HO^•^ with the guanine moiety is strictly dependent on the molecular context. The chemistry changes completely on going from the free base (Gua) to the nucleoside (dGuo) and from dGuo to DNA or the G-quadruplex, as we described herein. Several authors have often reported the calculations of HO^•^ with Gua for introducing the DNA damage, and this caused confusion in such a multidisciplinary field.

The two faces of the G^•^ radical have been reproduced by various methods and at different levels of complexity. The paradigm of one-electron-oxidized guanosines formation under the one-electron reduction of 8-bromo-guanosines has been the driving force to obtain the two faces of G^•^, i.e., G(N2-H)^•^ and G(N1-H)^•^, and their connection via tautomerization. This allowed the revision of the reaction of the HO^•^ radical with guanosine derivatives, where the main path is the H-atom abstraction from the exocyclic NH_2_ group. Both faces of the G^•^ radical have been invoked in order to interpret the time-resolved spectroscopies at ds-ODNs, DNA, or G-quadruplexes ODNs and to associate them to 8-oxo-G yields. This review presents a reasonable overview of the G^•^ structure depending on molecular context and behavior.

The end-products (or lesions) of guanine DNA have been identified and successfully connected to the various mechanistic steps. Now, the comparative quantification of various purine lesions appears periodically and are related to health conditions. For example, 8-oxo-G and, to minor extent, Fapy-G have been measured in various human specimens and rationalized with various pathologies. Analogous data for the other lesions in Figure 15 are limited or absent. In the last two years, our group has been very active in the detection of 5′,8-cyclopurine lesions (including the 5′S-cdG and 5′R-cdG reported in Figure 15), providing results from cells, animals, and humans. Further perspectives of transdisciplinary research combining chemistry, mechanisms, and lesions formation can be foreseen in order to reach a satisfactory comprehension of the guanine radical involvement in DNA damage and human health.

## Data Availability

Not applicable.

## Appendix A

In this section, some fundamental information regarding the methods of generation of the reactive species reported in this review by ionizing radiation are given in order to facilitate the nonexpert readers. The reader is referred to the literature for further information [103,104].

Radiolysis of neutral water leads to the species e_aq_^−^, HO^•^, and H^•^, as shown in Reaction 1, where the values in parentheses represent the radiation chemical yields (*G*) in units of μmol J^−1^.

H_2_O + γ-irr → e_aq_^–^ (0.27), HO^•^ (0.28), H^•^ (0.062) (A1)

The reactions of HO^•^ with the substrates were studied by irradiating N_2_O-saturated solution (~0.02 M of N_2_O); under these conditions, e_aq_^−^ are converted into the HO^•^ radical via Reaction 2 (*k*_2_ = 9.1 × 10^9^ M^−1^ s^−1^), with *G*(HO^•^) = 0.55 μmol J^−1^; i.e., HO^•^ radicals and H^•^ atoms account for 90 and 10%, respectively, of the reactive species.

e_aq_^–^ + N_2_O + H_2_O → HO^–^ + N_2_ + HO^•^ (A2)

The reactions of e_aq_^–^ with the substrates were studied by irradiating deoxygenated solutions containing 0.25 M of *t*-BuOH. In the presence of *t*-BuOH, HO^•^ radicals are scavenged (Reaction 3, *k*_3_ = 6.0 × 10^8^ M^−1^ s^−1^), whereas H^•^ may be trapped only partially (Reaction 4, *k*_4_ = 1.1 × 10^6^ M^–1^ s^–1^).

HO^•^ + t-BuOH → (CH_3_)_2_C(OH)CH_2_^•^ + H_2_O (A3)

H^•^ + t-BuOH → (CH_3_)_2_C(OH)CH_2_^•^ + H_2_ (A4)

The sulfate radical anion, SO_4_^•−^, was generated by irradiating Ar-purged solutions containing 10 mM of peroxydisulfate S_2_O_8_^2−^ and ~0.1 M of *t*-BuOH as a HO^•^ radical scavenger (Reaction 5, *k*_5_ = 1.2 × 10^10^ M^−1^ s^−1^).

e_aq_^–^ + S_2_O_8_^2–^ → SO_4_^2–^ + SO_4_^•–^ (A5)

The dibromine radical anion, Br_2_^•−^, was generated by irradiating N_2_O-saturated solutions containing 0.1 M of KBr. Under these conditions, at pH 7, the HO^•^ radical is converted into Br_2_^•−^, through the process shown in Reaction 6 (*k*_6_ = 1.1 × 10^10^ M^−1^ s^−1^).

HO^•^ + 2Br^–^ → Br_2_^•–^ + HO^–^ (A6)
